# Inferring longitudinal patterns of group B Streptococcus colonization during pregnancy

**DOI:** 10.1016/j.isci.2023.107023

**Published:** 2023-06-08

**Authors:** Bronner P. Gonçalves, Onur Poyraz, Proma Paul, Joy E. Lawn

**Affiliations:** 1 Department of Infectious Disease Epidemiology, London School of Hygiene & Tropical Medicine, London, UK; 2 Maternal, Adolescent, Reproductive & Child Health Centre, London School of Hygiene & Tropical Medicine, London, UK; 3 Department of Comparative Biomedical Sciences, School of Veterinary Medicine, University of Surrey, Guildford, UK; 4 Department of Computer Science, Aalto University School of Science, Aalto, Finland

**Keywords:** Microbiology, Statistical computing

## Abstract

Maternal colonization by Group B Streptococcus (GBS) can lead to severe infection in neonates and has also been associated with prematurity and stillbirth. Better quantitative understanding of the trajectories of GBS carriage during pregnancy is essential for the design of informative epidemiological studies. Here, we describe analyses of published longitudinal data using Bayesian hidden Markov models, which involve the estimation of parameters related to the succession of latent states (infection status) and observations (culture positivity). In addition to quantifying infection acquisition and clearance probabilities, the statistical approach also suggests that for some longitudinal patterns of culture results, pregnant women were likely to have been GBS-colonized despite a negative diagnostic result. We believe this method, if used in future longitudinal studies of maternal GBS colonization, would improve our understanding of the pathologies linked to this bacterium and could also inform maternal GBS vaccine trial design.

## Highlights

• Analyses of longitudinal GBS data that account for imperfect detection
• The approach allowed quantification of GBS acquisition and clearance rates
• Some pregnant women are likely colonized despite negative culture results
• The approach will be useful in future epidemiological studies on GBS carriage

## Graphical abstract

**Figure:**
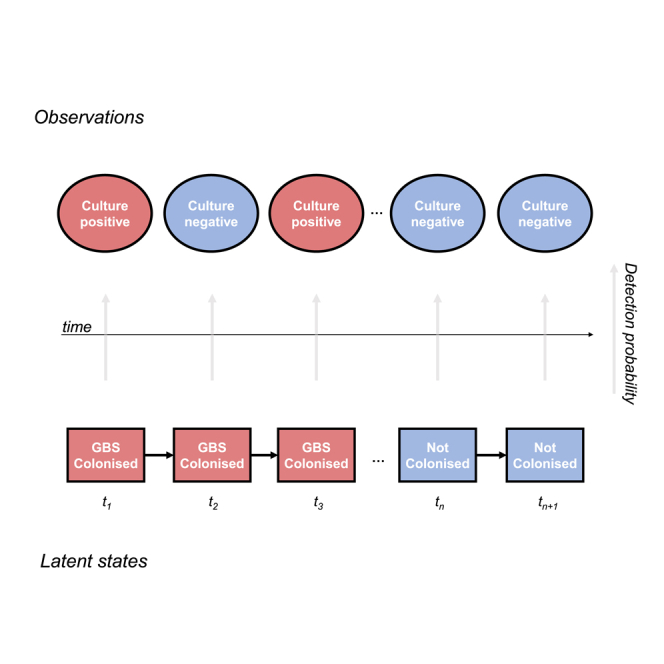

## Introduction

Both pregnant women and young infants are at risk of developing severe disease caused by Group B Streptococcus (GBS).^1^ In addition to the direct morbidity and mortality linked to the invasive presentation of GBS infection, GBS carriage during pregnancy has also been associated with an increased risk of preterm birth,^2^ which itself is linked to poor outcomes for newborns,^3^ and identified as a cause of stillbirth.^4^ Although maternal recto-vaginal colonization by this bacterium is believed to be the common component of the different mechanisms that lead to these pathological conditions,^5^ most epidemiological studies on GBS and pregnancy report prevalence, and only a few previous studies were designed to assess the longitudinal patterns of GBS carriage^6^^,^^7^^,^^8^^,^^9^^,^^10^ (see also Table S1). Analyses that coherently deconstruct GBS colonization data by separately estimating incidence and duration, i.e., the determinants of prevalence, of GBS carriage in pregnant women would not only improve our understanding of how GBS colonization increases the risk of prematurity and stillbirth but could also be used in the development and optimization of preventative approaches, for example regarding the timing of microbiological testing for culture result-based antibiotic prophylaxis. Furthermore, reliable quantification of GBS acquisition and clearance rates would be valuable in assessments of the effect of immunity, natural or vaccine-induced, on GBS colonization.

In this study, we re-analyze published data on GBS carriage in pregnant women. We use hidden Markov models that estimate transition probabilities between latent states, here related to GBS colonization, and account for the imperfect sensitivity of diagnostic methods in linking these latent states to observations (microbiological culture results).

## Results

### Data

**Figure 1:**
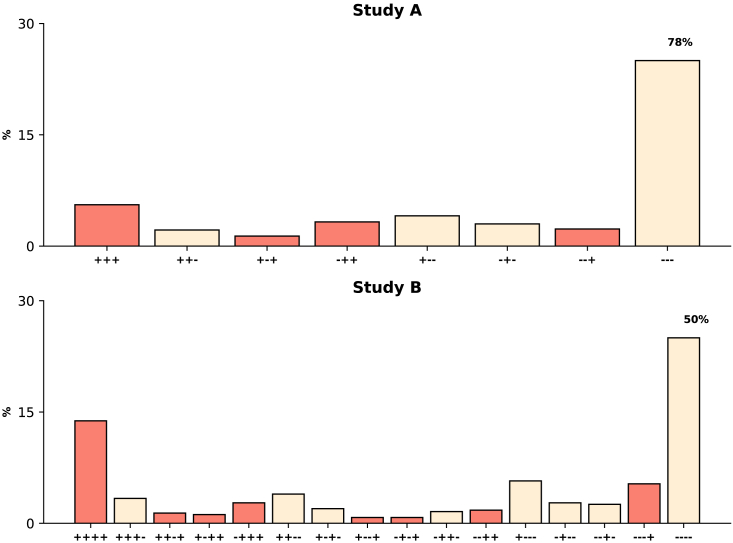
Distributions of the sequences of GBS detection results The y axes correspond to the percentages of study participants with each possible longitudinal pattern (x axes). Red bars represent frequencies of sequences in which there was evidence of GBS colonization in the final visit analyzed. Note that in each panel the upper limit of the y axis was set to 30%, and the frequency of the sequence with negative culture results in all visits is presented as text above the corresponding bar.

We performed a systematic literature search and identified two studies with data on repeated assessments of GBS colonization during pregnancy; information on the frequencies of different sequences of diagnostic results was abstracted from tables in the original publications and analyzed here. The first study, by Goodman et al.^7^ henceforth *Study A*, reported complete (all four study visits) longitudinal data for 735 pregnant women; only the first three study visits for each participant were included in this analysis as the time interval between the third visit and the fourth visit, at delivery, might have been variable. The second study, by Kwatra et al.^10^ will be referred to as *Study B*; that study described longitudinal patterns of detectable GBS carriage for 507 pregnant women. Figure 1 presents the frequencies of the possible sequences of microbiological results for both studies. Additional information on these studies is included in the STAR Methods section.

### Incidence and clearance of GBS carriage during pregnancy

Hidden Markov models (HMM) were used to describe GBS colonization dynamics during pregnancy. This statistical method is widely used to investigate time series, and explicitly model both (i) the transition between unobserved latent states, here the infection status, and (ii) the succession of observations, which here are dependent on the sensitivity and specificity of the culture method and correspond to microbiological results. A Bayesian approach was used to fit HMM to data^11^^,^^12^^,^^13^ from each study separately.

Figure S1 presents posterior predictive checks^14^ for the observed quantities, i.e., frequencies of the sequences of culture results; in performing that analysis, we accounted for the sequential nature of the data generating process (see also STAR Methods section). Although broadly consistent with the frequencies in the studies, the predictive checks suggest a slight misestimation of the occurrence of some longitudinal patterns in *Study B*. The estimated probability (posterior median and 95% posterior interval) of GBS carrier status in the first visit was 0.16 (0.13–0.20) for *Study A* and 0.35 (0.30–0.40) for *Study B*. Posterior estimates, in percentage, of the microbiological culture sensitivity for the two studies were 79% (73–85) and 86% (81–90) (posterior medians and 95% posterior intervals; *Study A* and *Study B*, respectively); sensitivity analyses using different prior assumptions for this parameter are presented in Table S4. The estimated risks, presented as proportions, for non-carriers of becoming GBS carriers between visits, i.e., the probabilities of transitioning from non-infected to infected, were 0.03 (0.01–0.05) and 0.05 (0.03–0.08) in *Study A* and *Study B*, respectively. The probability of clearance of GBS colonization between two consecutive visits was 0.20 (0.11–0.29) in *Study A* and 0.16 (0.11–0.21) in *Study B*; note that the typical time interval between successive visits was not the same in the two studies.

#### Likely trajectories of bacterial carriage

**Figure 2:**
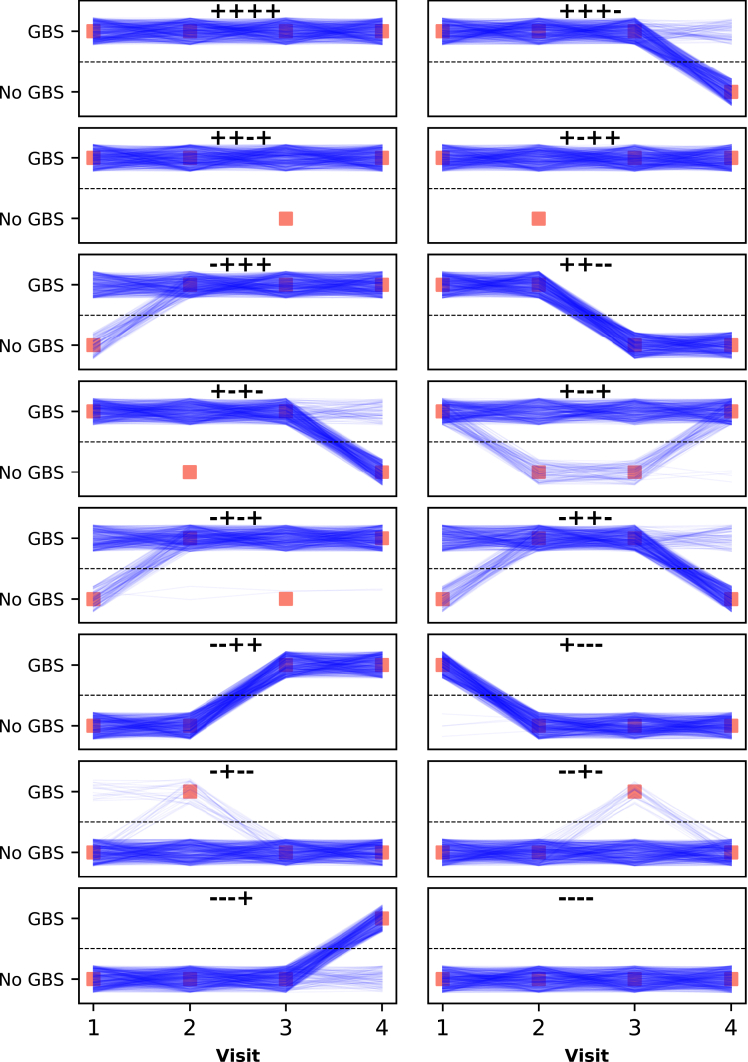
Likely GBS carriage trajectories for *Study B* For ease of visualization, 500 of the 4,000 posterior samples are presented. Each panel represents both the sequence of microbiological results, as red squares and title, and the estimated likely sequences of hidden states, i.e., carrier status (blue lines). Each posterior sample is represented by a blue line; uniformly distributed random values were added to y axis coordinates of individual trajectories to avoid superposition of lines. Figure S4 presents estimated likely trajectories from a model that uses a different prior assumption for assay sensitivity. As a discrete-time model was used, the lines are not intended to represent states between visits; rather they are used to indicate that the states in the same line were estimated using the same posterior sample, and thus jointly correspond to a trajectory.

Posterior parameter samples were used to identify the most likely successions of hidden states (carrier status and non-carrier status). In Figure 2, we present the sixteen possible observation patterns in *Study B*, i.e., the sixteen possible sequences of culture results with four visits, and estimated likely successions of latent states. For example, some study participants with GBS detected by culture in the first and fourth visits, but not in the second and third visits, might have been colonized throughout the study. In Figure S2, similar graphs are presented for all possible observation patterns in *Study A*.

#### Misclassification of the carrier status

**Figure 3:**
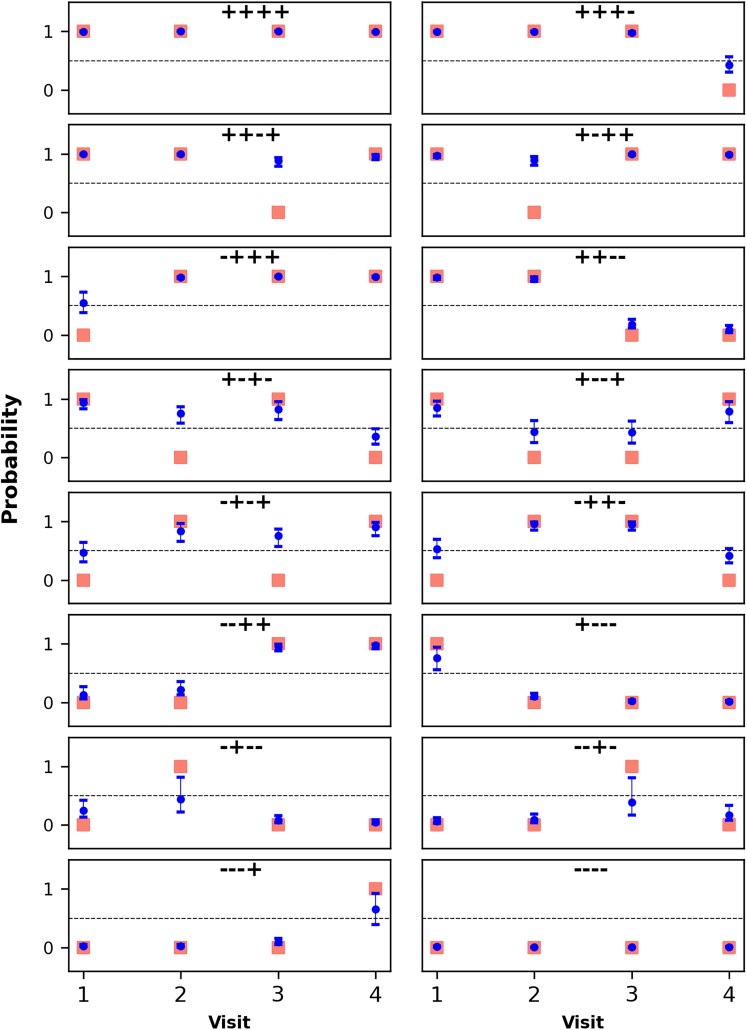
Probability of the GBS carrier state, i.e. probability of being truly infected, in each visit for *Study B* Individual panels represent possible sequences of diagnostic results. For each posterior sample, the forward-backward algorithm was used to estimate the probability of GBS carriage in each visit (x axes). The posterior median and 95% posterior interval of this probability are presented in blue; as in Figure 2, red squares represent observed microbiological results. Note that although 95% intervals are presented in all panels for all time points, some of the intervals are narrow. In this figure, the y axes represent both the two possible observations (“GBS” and “No GBS”) and the real-valued interval [0, 1].

We estimated the probability of the GBS carrier latent state in each visit using all evidence—by this, we mean that both the preceding and subsequent time points inform estimation, using the forward-backward algorithm, of the probability of being truly infected in a study visit. As can be observed in Figure 3 (*Study B*) and Figure S3 (*Study A*), the probability of GBS carriage during a visit with negative culture result was generally higher when the preceding and subsequent visits were GBS positive by culture, with estimated probabilities often above 0.5 for some of the possible sequences of observations (microbiological results).

## Discussion

Although the preventative strategy of antibiotic administration at delivery for women colonized by GBS or with risk factors has led to reductions in the incidence of GBS disease in many high-income countries,^15^^,^^16^^,^^17^ recent studies suggest that invasive GBS infection during early infancy remains a major cause of burden globally,^18^ in part due to long-term sequelae.^19^^,^^20^ Other pregnancy-associated conditions linked to GBS, including stillbirth, preterm birth, and maternal disease, also represent a public health problem in all regions. A key step in these disease processes is the recto-vaginal colonization by GBS bacteria. To gain new insights on GBS colonization, we re-analyze published data that describe how GBS carriage changes during pregnancy using a statistical method that accounts for both the longitudinal nature of data and the imperfect sensitivity of diagnostic assays.

For this analysis, we identified two studies performed more than 10 years apart and with different frequencies of GBS colonization (12.1–14% in *Study A*, and 28.4–33% in *Study B*; the ranges represent frequencies in different visits).^7^^,^^10^ The sensitivity of the culture method used in each study was estimated, being slightly higher in the more recent study (*Study B*) and only marginally affected by prior assumptions and estimation procedure (see Tables S2 and S4). Although only a small proportion of pregnant women was estimated to become GBS-colonized between consecutive visits (posterior medians 0.03 and 0.05 in Study A and Study B, respectively), the probability of GBS clearance was relatively low (posterior median 0.20 in *Study A* and 0.16 in *Study B*), explaining the patterns in Figure 2, when applying an algorithm to identify the most likely succession of hidden states. Note that the estimated probability of GBS colonization clearance in *Study B* is different from that reported in the original study, possibly because the current model accounts for imperfect assay sensitivity.

One objective of our analysis was the estimation of the most likely latent state in each study visit given information on all visits, which could be useful when defining GBS-related exposures or outcomes in future epidemiological studies. In Figures 3 and S3, we observe that in some study visits with negative culture result it was likely that the participant was colonized with GBS. These findings are consistent with earlier observations, including of a Danish study that followed women during and after pregnancy and found that some GBS carriers had fluctuating culture positivity.^8^ This analytical approach, which uses the entire data to infer the carrier status, potentially reducing misclassification, could be valuable for example in studies that aim to quantify the association between immune responses and GBS carriage (study outcome) during pregnancy, as well as epidemiological studies on the effect of GBS colonization (time-varying study exposure) on maternal outcomes (e.g. clinical disease) and neonatal outcomes (e.g. preterm birth or early onset invasive infection and associated long-term consequences).

We believe the use of models similar to those presented here will provide concrete insights on GBS epidemiology and policy. Indeed, even in settings with high intrapartum antibiotic prophylaxis coverage, early onset GBS disease still occurs, and several factors contribute to the suboptimal effectiveness of this approach,^21^^,^^22^^,^^23^^,^^24^ including: missed antenatal microbiological screening, errors in processing and communication of GBS screening results, failure to administer intrapartum antibiotics despite the presence of risk factors for early onset disease in women who tested negative at screening. Importantly, the limited sensitivity of diagnostic assays is believed to be a key determinant of the persisting incidence of invasive disease during the first week of life. Furthermore, pregnant women who have truly negative culture results at screening can become infected before delivery, and do not receive appropriate antibiotic prophylaxis. Previous studies also reported that women colonized with GBS at antenatal screening might have negative culture results at delivery,^25^ which suggests that for some mothers intrapartum prophylaxis might be administered even if their children are not necessarily at increased risk of GBS disease. Better quantification of GBS colonization incidence and duration, especially during the third trimester of pregnancy, is needed.

HMMs, which have been applied to the study of other clinical conditions but to our knowledge not of GBS, could be used for assessments of modified or novel preventative strategies. Although our analysis estimated GBS acquisition and clearance probabilities in two studies accounting for imperfect sensitivity of diagnostics, the relevance of our findings would be increased if data were available from multiple epidemiological settings and that used recent diagnostic developments, i.e. polymerase chain reaction (PCR)-based methods. Prospective studies with more frequent sampling and that use, for comparative purposes, both culture and molecular biology techniques and assess markers of pathogenicity would allow a more complete characterization of GBS colonization dynamics; and the statistical approach used here would be a valuable tool to gain insights from these data, including into whether specific patterns of carriage are associated with a higher risk of neonatal disease. Note that although more sensitive diagnostic methods would reduce the probability of a false-negative result at GBS screening, these methods would likely more often capture colonizations with light bacterial load, which, as suggested in a previous systematic review,^26^ are associated with a lower risk of neonatal colonization by GBS. A particularly useful extension of our work would combine longitudinal microbiological and immunological data to improve our understanding of the effect of naturally acquired or vaccine-induced immunity on GBS colonization acquisition and clearance^27^^,^^28^; this would also be informative for the design of trials of maternal GBS vaccines.

### Limitations of the study

This study used published aggregated data, and we were unable to assess the impact of individual characteristics on GBS carriage acquisition, clearance, and detection. Furthermore, only data from participants who completed the scheduled follow-up were included in this analysis; if GBS carriage in these participants differed from carriage in the population of pregnant women in the settings where the two studies were undertaken, parameter estimates reported here might not be applicable to the target populations of the original studies. Finally, our analysis did not account for the precise time intervals between consecutive visits, which might have varied non-negligibly between study participants.

## STAR★Methods

### Key resources table

**Table:** 

REAGENT or RESOURCE	SOURCE	IDENTIFIER
**Software and algorithms**		

Stan	The Stan Development Team	https://mc-stan.org/
HMMmcmc library	This paper	https://github.com/onurpoyraz/chmmMCMC
Python version 3.7	Python Software Foundation	https://www.python.org/

### Resource availability

#### Lead contact

Requests for further information should be directed to and will be fulfilled by the lead contact, Bronner Gonçalves (bronnergoncalves@gmail.com)

#### Materials availability

This study did not generate new unique reagents.

### Experimental model and study participant details

This study did not use experimental models. Data presented in peer-reviewed publications on GBS carriage in pregnant women were used for this analysis; the studies were performed in South Africa and in the United States. Additional information can be found below.

### Method details

#### Literature search

We performed a systematic literature search in PubMed using the expression “(pregnancy or pregnant) AND (longitudinal OR dynamics OR dynamic OR acquisition OR loss) AND (Group B Streptococcus OR GBS OR streptococcal)” to identify studies published after 1995 that reported longitudinal data on GBS carriage during pregnancy; these studies are listed Table S1 (see also Figure S5).

#### Data

In analyses presented in the results section, we used published data from two studies. These data were reported in tables of the manuscripts or the supplementary material files. In the first study,^7^ conducted by Goodman and colleagues in the United States, study participants were seen in the first trimester of gestation, at 26–28 weeks, 37 weeks, and delivery; lower vaginal and perianal samples were collected. In the second study,^10^ performed in South Africa, pregnant women were seen initially at week 20–25 of gestation and then at 5-week intervals (total of four visits), and lower vaginal and rectal swabs were used for microbiological testing. Details of the microbiological methods used in each study are described in the original publications.

### Quantification and statistical analysis

#### Statistical methods

We analysed data using first order discrete time HMMs, which have two key properties: 1) at each time point the latent state determines the probability distribution for the observations (i.e. observations are conditionally independent given latent states), which in this case can be seen as noisy outcomes of the latent states, and 2) the probabilities of transition from the current state to the possible states in the following time point do not depend on previous latent states (Markov property). Two processes are thus modelled, an observed and a hidden process. Note that in this manuscript the terms *hidden* and *latent* are interchangeable.

Below, we present the marginal likelihood of the HMM (Equation 1), using the same notation as in^12^^,^^29^; as explained by Leos-Barajas and Michelot, the use of the marginal likelihood, summing over the possible sequences of latent states (*s*_*t*_, with *t* representing different study time points), is necessary to fit the HMM using Stan software.

(Equation 1) $$L=\sum_{s_{1}}\sum_{s_{2}}..\;.\;\sum_{s_{T}}\delta_{s_{1}}\prod_{t=2}^{T}\gamma_{s_{t-1}{,s}_{t}}\prod_{t=1}^{T}f_{s_{t}}\left(y_{t}\right)$$

In the equation above, $\delta_{s_{1}}$ corresponds to the probability that the latent state at the start of the sequence is *s*_*1*_; and $\gamma_{s_{t-1}{,s}_{t}}$ represents state transition probabilities for two consecutive time points, and corresponds to entries in a, here two by two, transition matrix. The transition probabilities are assumed to be time-invariant.

In Equation 2, we define the probability distribution of observations given the latent state *s*_*t*_, $f_{s_{t}}\left(y_{t}\right)$, that was used in this analysis. As mentioned in the results section, the states in the model correspond to the infection status; *θ*_*j*_ represents the probability of a positive culture result when state *s*_*t*_ is *j*, and *y*_*t*_, culture results, with numerical values of zero or one.

(Equation 2) $$f_{s_{t}=j}\left(y_{t}\right)={\theta_{j}}^{y_{t}}{\left({1-\theta }_{j}\right)}^{1-y_{t}}$$

Model parameters include transition probabilities between non-carrier and carrier states, which correspond to incidence and clearance of GBS colonisation between study visits, and sensitivity and specificity of diagnostic methods used. The likelihood can be efficiently evaluated using the forward algorithm, that involves the recursively defined quantity^29^:

(Equation 3) $$\alpha_{t}\left(j\right)=\sum_{i=1}^{N}\alpha_{t-1}\left(i\right)\;\gamma_{i,j}\;f_{s_{t}=j}\left(y_{t}\right)$$

where $\gamma_{i,j}$ and $f_{s_{t}}\left(y_{t}\right)$ are defined above, and $\alpha_{1}\left(j\right)$ = $\delta_{s_{1}=j}f_{s_{1}=j}\left(y_{1}\right)$.

In the *Supplementary Appendix* (Figure S1), we present posterior predictive checks. Briefly, we used posterior samples of the initial probabilities of each state and of the transition probabilities to generate sequences of latent states (infection status) for each set of posterior samples; posterior samples of GBS culture detection probability were then used to generate datasets with the same sizes as those of the original studies that included simulated sequences of culture results for each participant.

Furthermore, we used the Viterbi algorithm to identify the most likely sequence of hidden states, i.e. the trajectories of bacterial carriage, for study participants, given the observed data. In other words, the Viterbi algorithm, or global state decoding, identifies the trajectory satisfying:

(Equation 4) $$s^{*}=\underset{s_{1:T}}{\mathrm{argmax}}p\left(s_{1:T}\;|\;y_{1:T}\right)$$

where ***s***_***1:T***_ represents the vector of latent states in a trajectory, and ***y***_***1:T***_, the data vector.

The forward-backward algorithm, also called smoothing or local state decoding, was used to identify the most likely state in each study visit. Briefly, this algorithm uses information on all study visits when estimating the probability of each latent state during a specific time point – i.e. implementation involves quantities dependent both on past visits and on future visits. The algorithm estimates $p\left(s_{t}\;|\;y_{1:T}\right)$, where ***y***_***1:T***_ is as defined above, and *s*_*t*_ is the latent state in time point *t*. Detailed information on the methodology of and code for these two well-described algorithms can be found in the following references.^12^^,^^13^^,^^29^^,^^30^

Data processing and visualisation were performed using Pandas and matplotlib libraries in Python (version 3.7). Bayesian analyses were performed with both PyStan in Python^31^ and the HMMmcmc library in R; the latter is available in https://github.com/onurpoyraz/chmmMCMC. The two libraries use different estimation procedures; additional information on the algorithm used in the HMMmcmc library is available in.^11^ Results using these methods were similar and are compared in the *Supplementary Appendix* (Table S2). Assumptions on prior distributions are described in Table S3; estimations from sensitivity analyses are shown in Table S4.

## Data Availability

• Data: Data are available as tables in the original publications cited in the manuscript. • Code: PyStan code is available in (https://github.com/BronnerG/HMM_GBS); and the HMMmcmc library is available in https://github.com/onurpoyraz/chmmMCMC. • Any additional information required to reanalyze the data reported in this paper is available from the lead contact upon request.
