# Supplementary material for: Inferring longitudinal patterns of group B Streptococcus colonization during pregnancy
Source: iScience. 2023 Jun 8;26(7):107023. doi: 10.1016/j.isci.2023.107023 (PMC10391671; doi:10.1016/j.isci.2023.107023)
Supplement: Document S1. Figures S1–S5 and Tables S1–S4 [file mmc1.pdf]

iScience, Volume *26*

Supplemental information

Inferring longitudinal patterns of group B Streptococcus colonization during pregnancy

Bronner P. Gonçalves, Onur Poyraz, Proma Paul, and Joy E. Lawn

Table S1. Publications with longitudinal data (at least 3 samples) on GBS carriage during pregnancy. Only studies published after 1995 were reviewed. The two studies whose data were re-analysed in this manuscript are not included in this table, as they are described both in the *Results* and *Methods* sections. **Table S1** is related to **Figure 1**, which shows aggregated data from the two studies analysed.

Study	Sample size	Number/frequency of study visits	Samples	Summary
Hansen et al. Dynamics of Streptococcus agalactiae Colonization in Women during and after Pregnancy and in Their Infants[1]	77 pregnant women (from week 16)	3 or more samples	Vaginal and anorectal swabs	28% of pregnant women were persistent carriers, and 19% were intermittent carriers.
Mavenyengwa et al. Group B Streptococcus colonization during pregnancy and maternal-fetal transmission in Zimbabwe[2]	1,037 pregnant women (from early second trimester; 623 with GBS colonisation data on the 3 visits)	3 samples	Lower vaginal and rectal samples	GBS colonisation was more frequently observed in pregnant women living rural compared to urban areas. Frequency of GBS colonisation was higher earlier during pregnancy but had a low positive predictive value for colonisation at delivery.
Foxman et al. Acquisition and Transmission of Group B Streptococcus during Pregnancy[3]	78 pregnant women (third trimester)	3 samples	Vaginal and rectal swabs	The study described transmission in couples with discordant GBS status. One-month GBS acquisition rate among women in their 3rd trimester was 4.7%.
Brzychczy-Włoch et al. Dynamics of colonization with group B streptococci in relation to normal flora in women during subsequent trimesters of pregnancy[4]	42 pregnant women (first trimester)	3 samples	Vaginal and rectal swabs	The study assessed differences in the composition of vaginal and rectal flora in pregnant women who were GBS-colonised versus pregnant women who were not GBS-colonised. GBS colonisation fluctuated and varied based on specimen site and trimester.

Table S2. Comparison of posterior means from analyses using the PyStan library (Python) and the HMMmcmc library (R). Note that the probabilities of positive culture result for the two latent states correspond to emission probabilities in HMM literature terminology. **Table S2** relates to **Figure 2** and **Figure 3**; both figures were created using posterior samples.

	**STUDY A**			
Parameters	PyStan library		HMMmcmc library	
	GBS carrier state		GBS carrier state	
Initial state probability	0.16		0.16	
	GBS carrier state	Non carrier state	GBS carrier state	Non carrier state
Probability of positive culture	0.79	0.01	0.77	0.02
	GBS carrier state	Non carrier state	GBS carrier state	Non carrier state
Transition probabilities				
from GBS carrier state	0.80	0.20	0.86	0.14
from Non carrier state	0.03	0.97	0.02	0.98
	**STUDY B**			
Parameters	PyStan library		HMMmcmc library	
	GBS carrier state		GBS carrier state	
Initial state probability	0.35		0.33	
	GBS carrier state	Non carrier state	GBS carrier state	Non carrier state
Probability of positive culture	0.86	0.03	0.89	0.05
	GBS carrier state	Non carrier state	GBS carrier state	Non carrier state
Transition probabilities				
from GBS carrier state	0.84	0.16	0.84	0.16
from Non carrier state	0.05	0.95	0.04	0.96

Table S3. Prior assumptions in the primary analysis, using the PyStan library (Python). The informative prior used for the probability of positive culture result, which corresponds to the test sensitivity when a participant is colonised by GBS, is consistent with estimates in the manuscript by Kwatra et al[5], and references therein. **Table S3** relates to **Figure 2** and **Figure 3**; both figures were created using posterior samples.

Parameters	**Prior assumptions**	
	GBS carrier state	
Initial state probability	Uniform(0,0.5)*	
	GBS carrier state	Non carrier state
Probability of positive culture	Beta(80,20)	Beta(1,99)
	GBS carrier state	Non carrier state
Transition probabilities		
from GBS carrier state	Dirichlet(2,2)	
from Non carrier state	Dirichlet(2,2)	

*constrained for identifiability

Table S4. Sensitivity analyses using different prior assumptions. Posterior estimates of parameters that are not included in the table were similar to estimates presented in the *Results* section. **Table S4** relates to **Figure 2** and **Figure 3**; both figures were created using posterior samples from the primary analysis. See also **Figure S4**.

Parameter modified	Prior used in primary analysis	Posterior median (95% interval)	Prior - sensitivity analysis	Posterior median (95% interval)
Probability of positive culture	Beta(80,20) (Carrier state)	Study A: 79 (72 – 85) Study B: 86 (81 – 90)	Beta(20,5) (Carrier state)	Study A: 78 (68 – 87) Study B: 88 (83 – 92)
Probability of positive culture	Beta(80,20) (Carrier state)	Study A: 79 (72 – 85) Study B: 86 (81 – 90)	Beta(40,10) (Carrier state)	Study A: 78 (70 – 86) Study B: 87 (82 – 91)
Probability of positive culture	Beta(80,20) (Carrier state)	Study A: 79 (72 – 85) Study B: 86 (81 – 90)	Beta(70,30) (Carrier state)	Study A: 72 (65 – 79) Study B: 82 (78 – 86)
Probability of positive culture	Beta(80,20) (Carrier state)	Study A: 79 (72 – 85) Study B: 86 (81 – 90)	Beta(90,10) (Carrier state)	Study A: 86 (79 – 92) Study B: 90 (85 – 93)

Figure S1. Posterior predictive checks. Posterior samples were used to generate datasets with sequences of culture results; additional information is presented in the *Methods* section. For all possible result sequences (x-axes), percentages of participants, observed (bars; red bars correspond to sequences with GBS detection in the final visit) or simulated (lines), with the corresponding pattern are shown in the y-axes. Note that lines were used to improve visualisation as they allow presentation in the same figure of the different distributions of a categorical quantity in each simulated dataset (here the categorical quantity of interest is the possible sequence of diagnostic results, which has 2^n strata, *n* being the number of visits). In other words, the lines are used because if instead different sets of bars were used for each simulated dataset it would not be possible to discriminate which bars represent the same simulation. The upper and bottom panels represent *Study A* and *Study B*, respectively. A total of 100 posterior samples were used to generate these panels. **Figure S1** relates to **Figure 1**, **Figure 2**, **Figure 3**.

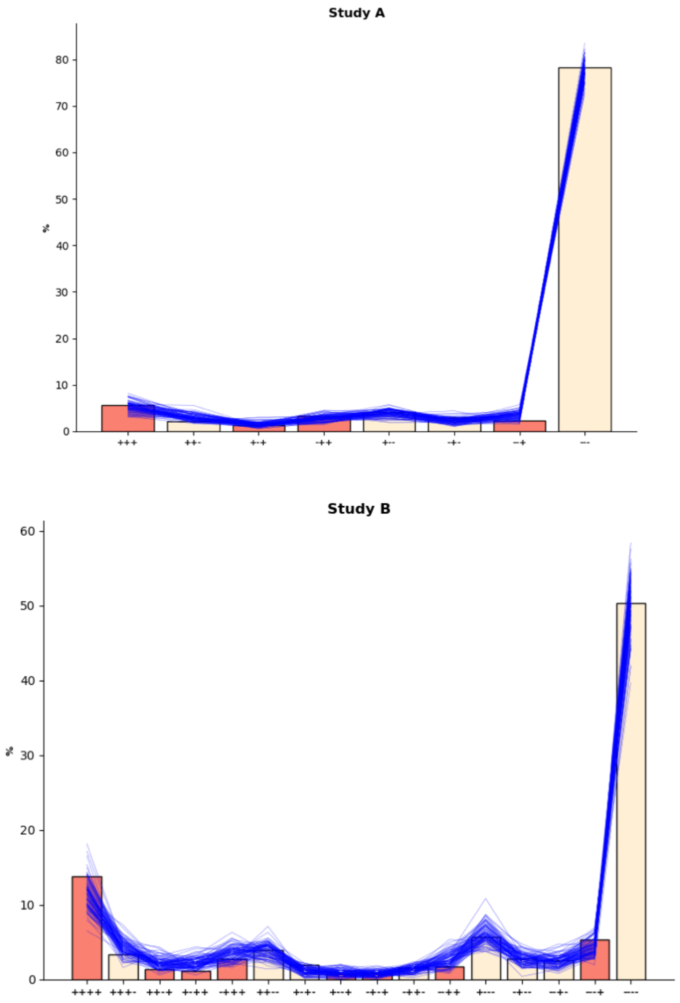

Figure S2. Likely GBS carriage trajectories for *Study A*. For ease of visualisation, 500 of the 4,000 posterior samples are presented. Each panel represents both the sequence of microbiological results, as red squares and title, and the estimated sequences of hidden states, i.e. carrier status (blue lines). Each posterior sample is represented by a blue line; uniformly distributed random values were added to the y-axis coordinates of individual trajectories to avoid superposition of lines. **Figure S2** is directly related to **Figure 2**.

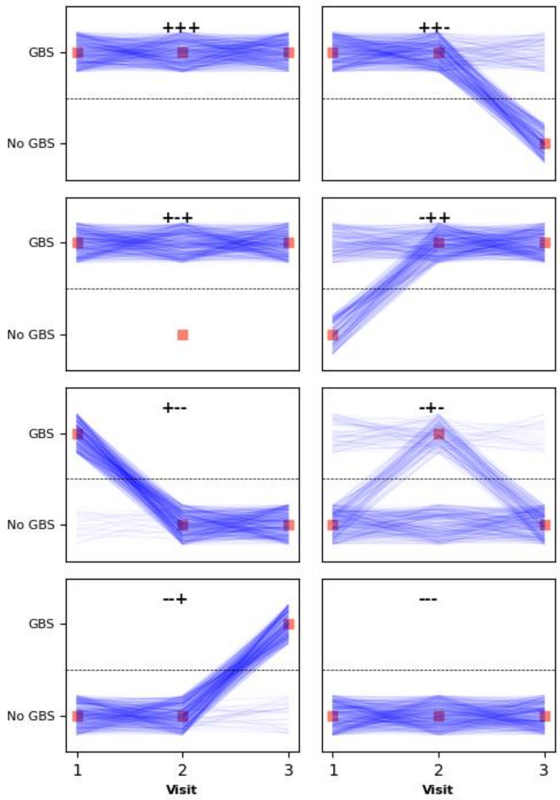

Figure S3. Probability of the GBS carrier state in each visit for *Study A*. Individual panels represent possible result sequences. For each posterior sample, the forward-backward algorithm was used to estimate the probability of GBS carriage in each visit (x-axes). The posterior median and 95% posterior interval of this probability are presented in blue; red squares represent microbiological results. Note that in this figure, the y-axes represent both possible observations (“GBS” and “No GBS”) and the real-valued interval [0, 1]. **Figure S3** is directly related to **Figure 3**.

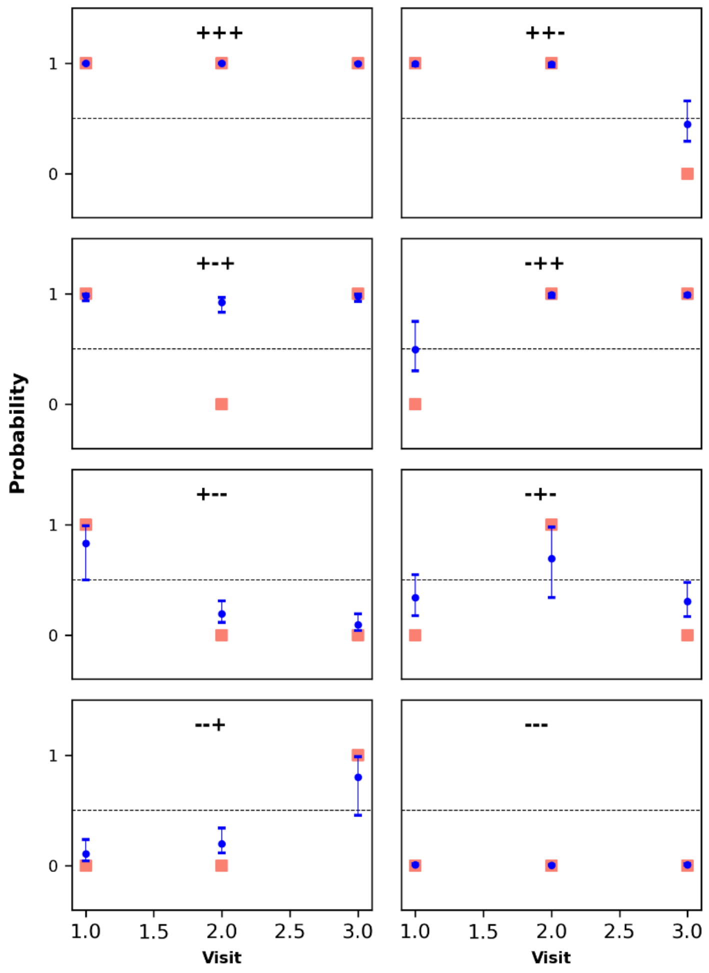

Figure S4. This figure is related and similar to **Figure 2** but presents likely GBS colonisation trajectories when using the following prior distribution for the assay sensitivity: Beta(20, 5).

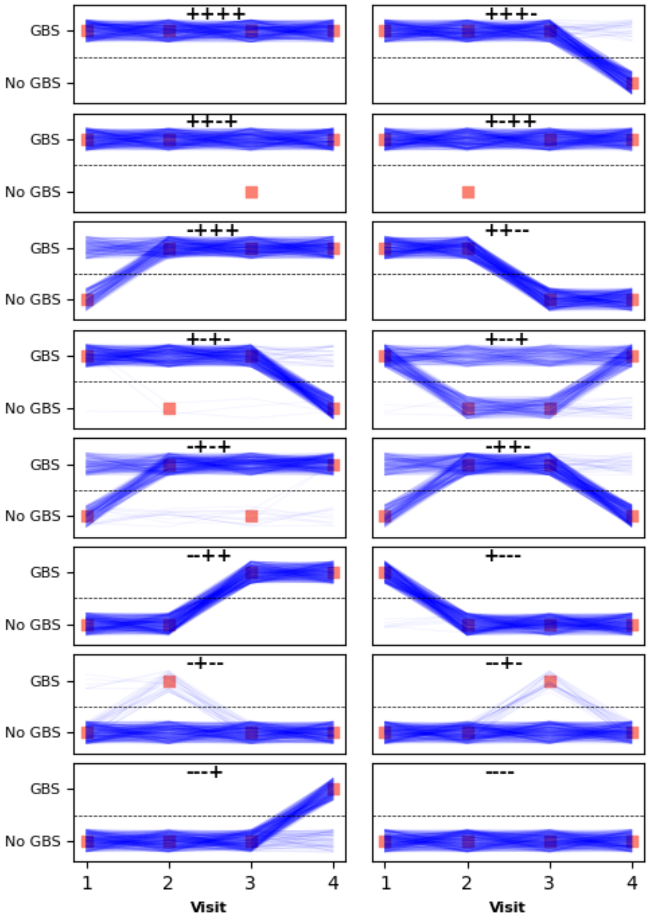

Figure S5. Description of literature search. **Figure S5** relates to **Figure 1**, where aggregated data from the two studies analysed are presented.

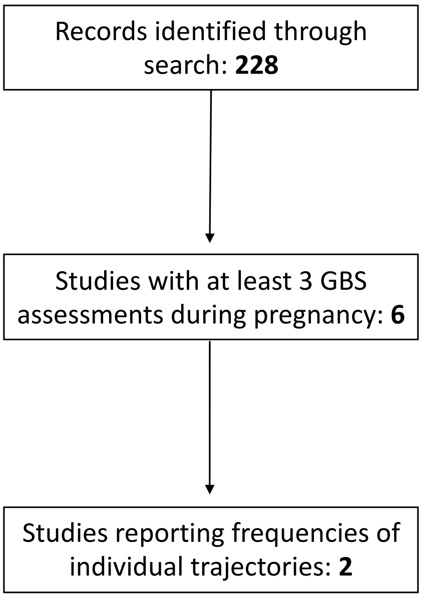

References

1. Hansen, S.M., Uldbjerg, N., Kilian, M., and Sorensen, U.B. (2004). Dynamics of Streptococcus agalactiae colonization in women during and after pregnancy and in their infants. J Clin Microbiol *42*, 83-89. 10.1128/JCM.42.1.83-89.2004.
2. Mavenyengwa, R.T., Afset, J.E., Schei, B., Berg, S., Caspersen, T., Bergseng, H., and Moyo, S.R. (2010). Group B Streptococcus colonization during pregnancy and maternal-fetal transmission in Zimbabwe. Acta Obstet Gynecol Scand *89*, 250-255. 10.3109/00016340903398029.
3. Foxman, B., de Azevedo, C.L., Buxton, M., DeBusscher, J., Pillai, P., De Carvalho, N.S., and Barbosa-Cesnik, C. (2008). Acquisition and transmission of group B Streptococcus during pregnancy. J Infect Dis *198*, 1375-1378. 10.1086/592221.
4. Brzychczy-Wloch, M., Pabian, W., Majewska, E., Zuk, M.G., Kielbik, J., Gosiewski, T., and Bulanda, M.G. (2014). Dynamics of colonization with group B streptococci in relation to normal flora in women during subsequent trimesters of pregnancy. New Microbiol *37*, 307-319.
5. Kwatra, G., Madhi, S.A., Cutland, C.L., Buchmann, E.J., and Adrian, P.V. (2013). Evaluation of Trans-Vag broth, colistin-nalidixic agar, and CHROMagar StrepB for detection of group B Streptococcus in vaginal and rectal swabs from pregnant women in South Africa. J Clin Microbiol *51*, 2515-2519. 10.1128/JCM.00251-13.
